# Klotho Protects Dopaminergic Neuron Oxidant-Induced Degeneration by Modulating ASK1 and p38 MAPK Signaling Pathways

**DOI:** 10.1371/journal.pone.0139914

**Published:** 2015-10-09

**Authors:** Reynolds K. Brobey, Dwight German, Patricia K. Sonsalla, Prem Gurnani, Johanne Pastor, C-C Hsieh, John Papaconstantinou, Philip P. Foster, Makoto Kuro-o, Kevin P. Rosenblatt

**Affiliations:** 1 Centers for Proteomics and Systems Biology, the Brown Foundation Institute of Molecular Medicine, UTHealth Medical School, 1825 Pressler Street, Houston, Texas, United States of America; 2 Division of Oncology, Department of Internal Medicine, UTHealth Medical School, 6410 Fannin, UTPB Suite 722, Houston, Texas, United States of America; 3 Department of Psychiatry, the University of Texas Southwestern Medical Center, 5323 Harry Hines Blvd., Dallas, Texas, United States of America; 4 Department of Neurology, UMDNJ Robert Wood Johnson Medical Center, Piscataway, New Jersey, United States of America; 5 Companion Dx Reference Lab, LLC, 10301 Stella Link Rd., Suite C, Houston, Texas, United States of America; 6 Department of Biochemistry and Molecular Biology, University of Texas Medical Branch, Galveston, Texas, United States of America; 7 Department of NanoMedicine and Biomedical Engineering, MD Anderson Cancer Center Bldg-3SCRB, 1881 East Road, Houston, Texas, United States of America; 8 Division of Pulmonary Medicine, Department of Internal Medicine, 6431 Fannin, MSB 1.274, Houston, Texas, United States of America; 9 Department of Pathology, Center for Mineral Metabolism, University of Texas Southwestern Medical Center, 5323 Harry Hines Blvd., Dallas, Texas, United States of America; 10 Center for Molecular Medicine, Jichi Medical University, 3311–1 Yakushiji, Shimotsuke, Tochigi, Japan; McGill University Department of Neurology and Neurosurgery, CANADA

## Abstract

Klotho transgenic mice exhibit resistance to oxidative stress as measured by their urinal levels of 8-hydroxy-2-deoxyguanosine, albeit this anti-oxidant defense mechanism has not been locally investigated in the brain. Here, we tested the hypothesis that the reactive oxygen species (ROS)-sensitive apoptosis signal-regulating kinase 1 (ASK1)/p38 MAPK pathway regulates stress levels in the brain of these mice and showed that: 1) the ratio of free ASK1 to thioredoxin (Trx)-bound ASK1 is relatively lower in the transgenic brain whereas the reverse is true for the Klotho knockout mice; 2) the reduced p38 activation level in the transgene corresponds to higher level of ASK1-bound Trx, while the KO mice showed elevated p38 activation and lower level of–bound Trx; and 3) that 14-3-3ζ is hyper phosphorylated (Ser-58) in the transgene which correlated with increased monomer forms. In addition, we evaluated the *in vivo* robustness of the protection by challenging the brains of Klotho transgenic mice with a neurotoxin, MPTP and analyzed for residual neuron numbers and integrity in the substantia nigra pars compacta. Our results show that Klotho overexpression significantly protects dopaminergic neurons against oxidative damage, partly by modulating p38 MAPK activation level. Our data highlight the importance of ASK1/p38 MAPK pathway in the brain and identify Klotho as a possible anti-oxidant effector.

## Introduction

Depletion of the *klotho* gene shortens lifespan and this has been documented in mammals, and in lower organisms [1,2]. In both cases, elevated oxidative stress is a major contributing factor for the aging-related phenotype. By contrast, overexpressing the *klotho* gene in mice extends lifespan with a concomitant lower level of oxidative stress [2,3].

We had shown that transgenic mice overexpressing the *klotho* gene survived a challenge with lethal doses of paraquat, an herbicide toxin that generates high levels of superoxide [3]. Further analysis showed that the mice urinary levels of 8-hydroxyguanosine caused by oxidant-induced mitochondrial DNA damage were significantly reduced [3]. Moreover, the secreted Klotho produced as recombinant protein was able to suppress paraquat-induced oxidative stress in CHO and Hela cells when added exogenously in culture. These observations suggest that reactive oxygen species (ROS)-sensitive signaling events operate in stress pathways affected by Klotho.

Endogenous ROS produced by mitochondrial electron transport chain (ETC) dysfunction activates the p38 MAPK pathway, which is a major stress-regulator, and therefore, a key contributor to stress-associated aging disorders [4,5]. This pathway is activated through the apoptosis signal-regulating kinase 1 (ASK1) signaling complex. We recently reported that the ASK1 signaling complex regulates p38 activity in the livers of Klotho overexpressing and Klotho deficient mouse [6]. If identified, the presence of a brain in situ antioxidant would emerge as a powerful factor potentially mitigating neurodegeneration since the antioxidant system was previously presumed underactive in the brain [7,8].

Here, we tested the hypothesis that the reactive oxygen species (ROS)-sensitive apoptosis signal-regulating kinase 1 (ASK1)/p38 MAPK regulates stress levels in the brain of these mice and showed that: 1) the ratio of free ASK1 to thioredoxin (Trx)-bound ASK1 is relatively lower in the transgenic brain whereas the reverse is true for the Klotho knockout mice; 2) the reduced p38 activation level in the transgene corresponds to higher level of ASK1-bound Trx, while the KO mice showed elevated p38 activation and lower level of–bound Trx, and 3) that 14-3-3ζ is hyper phosphorylated (Ser-58) in the transgene which correlated with increased monomer forms.

## Methods

### Animals

The generation of Klotho knockout mice (*klkl*) and transgenic mice that overexpress Klotho (*EFmKL46* or *EFmKL48*) has been described previously [2]. Mice employed in this study were maintained under extreme hygienic conditions. We followed the IACUC guidelines and the protocol was approved by the Institutional Animal Care and Research Advisory Committee of the University of Texas System.

### MPTP administration of mice

Age- and sex-matched *EFmKL46* or *EFmKL48*, *klkl* and their wild-type littermates (C57BL6 strain) (n = 5–7) were administered with MPTP (20 mg/kg) or vehicle by subcutaneous injection. The mice were euthanized 7 days after the injection by CO_2_ inhalation according to procedures established by the University of Texas Care and Use Committee and consistent with the recommendations of the Panel on Euthanasia of the American Veterinary Medical Association. Various tissues/organs were harvested, sliced, and immediately kept under liquid nitrogen atmosphere until use.

### Substantia nigra extraction and p38 MAPK assay on nitrocellulose array slides

Procedures used here are based on previous reports [9–11]. Briefly, frozen sections of Klotho mouse brain tissues were made onto Jung Woo slides (JungWoo International Co., Seoul, Korea). Substantia nigra regions were selectively procured using laser microdissection instrument (ION LMD; JungWoo International Co.). Tissue lysates were prepared from the microdissected material using published protocol [9] and arrayed in serial dilutions in triplicates onto nitrocellulose-coated FAST® glass slides (Whatman, Sanford, ME). Approximately 1.5 nL per spot was arrayed using the SpotArray 24 Microarray Printing System (PerkinElmer Inc., Waltham MA) and slides were stored at -20°C under desiccated environment or were used same day. Following spot array, slides were prepared for immunodetection as follows. Arrays were incubated for 15 min in 1X Reblot buffer (Chemicon, Temecula, CA) and washed twice for 5 min in Ca^2+^/Mg^2+^-free PBS (DPBS, GIBCO-Invitrogen). Washed slides were then blocked for at least 2 h in DPBS, supplemented with 2% I-Block^TM^ powder (Applied Biosystems, Foster City, CA) and 0.1% Tween-20. Further washes followed, and slides were finally air dried and array surfaces exposed to phospho-p38 MAPK antibody, followed with biotinyl tyramide amplification procedure. Thereafter, 20 nM Qdot® 655-streptavidin reagents (Life Technologies, Carlsbard, CA) were incubated on the array. Imaging was done with the ScanArray^TM^ (Perkin Elmer) and phosphorylated signal intensity was normalized with signal of replicate slides stained with fluorescent protein dye SyproRuby (Life Technologies).

### Protein preparation

Mouse brain lysates were prepared as follows: Approximately one gram (1g) of frozen brain tissue was cut into slices and homogenized on ice in 2 ml of commercially available protein extraction buffer (Active Motive, Carlsbad, CA), supplemented with protease and phosphatase inhibitors. Total homogenate was centrifuged at 10,000 rpm for 10 min using an Eppendorff refrigerated microcentrifuge, and the supernatant was saved in a fresh tube. The pellet fraction was extracted in a similar buffer to that above (but with ~10-fold increase in detergent) for an additional 30 min along with repeated vortexing. This extract was re-centrifuged at 14,000 rpm for 10 min and the supernatant fraction then combined with the first one above, divided into aliquots and kept at -80°C until needed. The protein concentration of the samples was determined using Bradford Assay Kit (Bio-Rad, Hercules, CA).

### Proteomics studies by 2D-GE

Two-dimensional gel electrophoresis **(**2D-GE) of brain lysates derived from Klotho mice was performed using the IPGphore isoelectric focusing (IEF) system (GE Healthcare). Briefly, clear lysates were cleansed by precipitation using the ProteoExtract Protein Precipitation Kit (EMD Biosciences) and reconstituted in lysis buffer (8 M urea, 2 M thiourea, 4% 3-[(3-Cholamidopropyl)dimethylammonio]-1-propanesulfonate (CHAPS), 2% dithiothreitol (DTT), x1 protease inhibitor cocktail) supplemented with 50 mM NaF/2 mM Na-orthovanadate. Upon incubation at room temperature for 30 min, protein samples were further diluted 3–4 fold (0.75 μg/μl) with rehydration buffer (9 M urea, 1 M thiourea, 2% CHAPS, 1% DTT, 0.2% ampholytes, pH 3–10) containing trace amounts of bromophenol blue. A 200 μl volume, (150 μg protein) was then applied onto the 11 cm immobilized pH gradient (IPG) strips (pH 4–7 or pH 3–10) and run at 50 V for 12 h. IEF was performed for a total of 64,000 Vh and focused strips were sequentially coated with SDS for 15 min in reducing buffer (50 mM Tris, pH 6.8, 6 M urea, 2% DTT, 2% SDS, 20% glycerol), and another 15 min in alkylating buffer (50 mM Tris, pH 8.8, 6 M urea, 2.5% iodoacetamide, 2% SDS, 20% glycerol). SDS-PAGE of the strips were run by established protocols using the criterion gels (Bio-Rad), and resolved proteins were first stained with Pro-Q Diamond (Life Technologies) for phosphoproteins, followed by SyproRuby staining of same gels for total protein assessment. Gel images were obtained with a Perkin-Elmer ProXPRESS Proteomic Imaging System (Boston, MA) and spots analysis was performed using the Progenesis SameSpots software (Newcastle upon Tyne, UK). Significant differential expression was held to a pairwise p-value of <0.05 and a fold-change of > 1.5.

### Immunoprecipitation, SDS-PAGE and Western blot

Immunoprecipitation (IP) assays were performed following established procedures but with modifications where necessary. Briefly, 200–400 μg proteins from brain tissue were incubated with 1–2 μg of the antibody for 6–7 h in IP buffer (20 mM Tris, pH 7.5, 150 mM NaCl, 2 mM EDTA, 1 mM EGTA), supplemented with protease/phosphatase inhibitor cocktail at 4°C, and 30 μl (bed volume) of protein A-conjugated agarose beads was added and incubated for additional 4 h. Following incubation, beads were washed five times with the same buffer, resuspended in 50 μl of sample loading buffer, and processed for conventional SDS-PAGE. Where no heating was necessary, samples were incubated with SDS buffer for 30 min at room temperature under reducing and/or non-reducing conditions. The resolved proteins in NuPAGE gels (4–12%) (Life Technologies), were electroblotted onto PVDF membranes (GE Healthcare) using the Trans-Blot Semi-Dry Transfer Cell system (Bio-Rad). Transfer was conducted at a constant current of 100 mA for 1 h. Blots were incubated with appropriate primary and secondary antibodies and protein signals were detected with the Novex^R^ ECL Western blotting detection reagents (Life Technologies). Where protein phosphorylation levels were measured, specific phosphorylated primary antibodies were used. Blots were then stripped using Restore Western Blot Stripping Buffer (Thermo Scientific/Pierce, Rockford, IL), and re-probed with the desired antibodies for total protein detection. The signal intensity of phosphorylated bands was normalized with that of the total protein and the average ratios were plotted with standard deviations.

### RNA extraction and PCR

Klotho gene expression in the various brain tissues was studied using both conventional RT-PCR and the RT^2^ qPCR assay system (SABiosciences, CA) in accordance with the manufacturer's recommendations on the Eppendorf Realplex 4S platform. Briefly, brain tissues from different regions of wild type C57BL/6 adult mouse were collected under RNase free conditions and total RNA was isolated using TRIZOL RNA isolation reagent (Life Technologies) and purified using PureLink Total RNA Purification System (Life Technologies). First-strand synthesis was oligo-d(T)-primed and Klotho and GAPDH were then amplified using specific primers. In the case of the qPCR, 1 μg of total RNA was treated with DNase and first strand cDNA synthesis was achieved using RT^2^ First Strand kit (SABiosciences). Relative mRNA expression was analyzed using actin or cyclophilin A primers as internal controls.

### Quantification of striatal DA and metabolites

Mice were euthanized by cervical dislocation. For neurochemical measurements, left and right striata were dissected, homogenized to 50 mg/ml in 0.1M phosphate buffer with 0.9% NaCl (PBS) and divided into two fractions. One fraction was diluted 1:5 with 0.25 M perchloric acid (PCA), homogenized by sonication and centrifuged at 17,000g for 7 min. The other fraction was used for determination of striatal TH content. Striatal DA was measured by HPLC with electrochemical detection as routinely performed in our laboratory [12,13].

### Measurement of striatal TH and MPP^+^ levels

TH were measured using an ELISA as reported by our laboratory previously [14]. MPP^+^ levels were measured in the striatum 7 hours after a single dose of MPTP (20 mg/kg) using methods previously described [15].

### Histology

Coronal sections were cut at 30 μm thickness. Consecutive sections were saved throughout the rostral-caudal extent of the substantia nigra. Every 6th section was immunostained with an antibody against TH (Protos Biotech Corp, NY; 1:4,000 diluted) to identify the substantia nigra (SN) DA somata.

### Stereology

Using the neuroanatomical landmarks for the SN [15], outlines of the SN were digitized and the number of neurons in the SN was estimated with the optical dissector stereological technique using StereoInvestigator Software (MicroBrightField, Inc.). A minimum of 250 neurons were counted. The total number of neurons, the volume of the nucleus and coefficient of error are calculated by the software. Such counting procedures routinely give coefficients of error of ≅0.06 (Schaefer). The individual performing the counting was blinded to the mouse group identification.

### Statistical analysis

Unless otherwise stated, all statistical analyses were done using 2-tailed t tests. Data were presented as means ± SEM. Analyses were performed for Klotho mutant verses wild type mice, to test the disparity in means between normalized phosphoprotein/protein signals at a significant level of 0.05. When the symbol (*) is applied it denotes statistically significant differences between the values portrayed by the error bars.

## Results

### The Trx/ASK1 complex level regulates p38 MAPK activity in the brains of Klotho mouse model of aging

**Fig 1:**
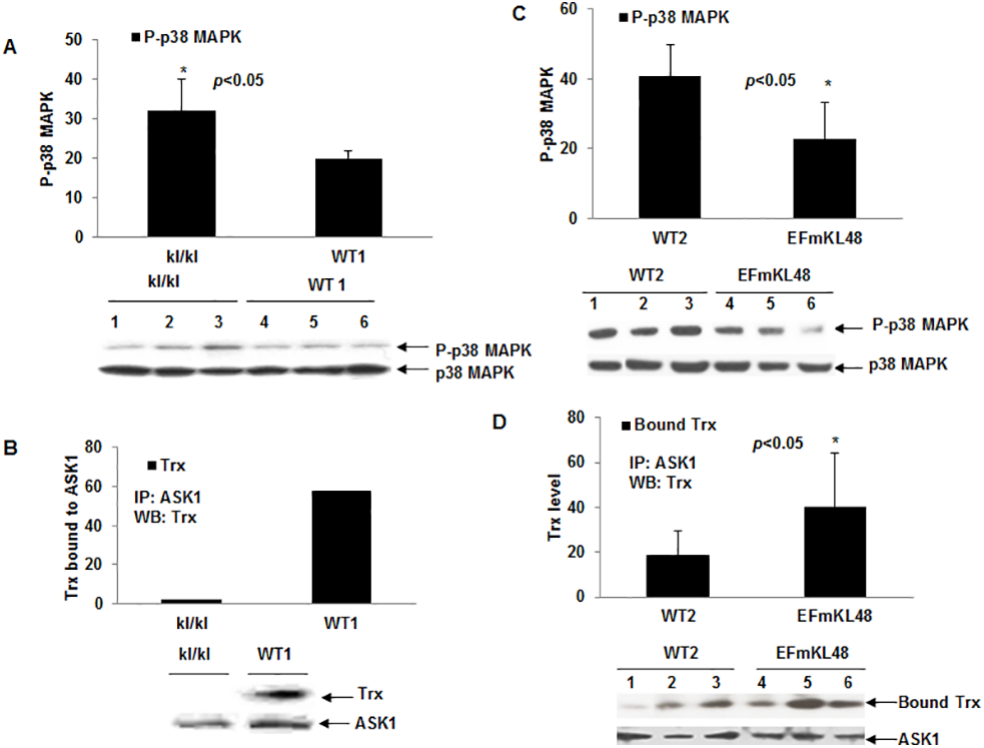
Levels of p38 MAPK activation and Trx/ASK1 complex formation in Klotho KO and Klotho transgenic mice. (A) Plots of phosphorylation levels of p38 MAPK in *kl/kl* mice and their WT littermates (WT1). Each bar represents average of three individual mouse and deviations are shown. *p< 0.05 between *kl/kl* and WT1 mice. (B) Levels of Trx bound to ASK1 in *kl/kl* and WT1 mice. ASK1 was IP with rabbit polyclonal ASK1 antibody; co-precipitated Trx was revealed by Western blot. Pooled total proteins from three mice were used for the IP. (C) Levels of p38 MAPK activation in EFmKL48 mice and their WT control (WT2). Each plot represents average of three mice and deviations are shown. *p< 0.05 between EFmKL48 and WT2 mice. (D) Levels of Trx bound to ASK1 in EFmKL48 mice and their WT2 control. *p< 0.05 between EFmKL48 mice and control WT2. Representative Western blot of the samples are shown below each plot. Digitized values of the WB signals were obtained with UN-SCAN-IT^TM^ software (Silk Scientific, Utah) and values are shown in S1 and S2 Tables. Phosphorylation levels were normalized by stripping same membrane and re-probing with the corresponding total antibody.

As Fig 1A indicates, the Klotho knockout mice (*klkl*) exhibited elevated basal levels of p38 MAPK phosphorylation (activation) (p<0.05), as compared to their WT littermates. Consequently, the endogenous Trx bound to ASK1 (i.e. Trx/ASK1 complex level) in the *klkl* was much lower than that of their WT littermates (Fig 1B), which is consistent with the elevated p38 MAPK activation due to increased free ASK1. By contrast, the *EFmKL48* mice displayed decreased basal p38 MAPK activation (p<0.05) (Fig 1C) and increased Trx bound to ASK1 (p<0.05) when compared with their WT littermates (Fig 1D). These results clearly show that Klotho overexpression in the brain produces effects that mitigate against p38 MAPK-mediated oxidative stress.

### 14-3-3 proteins are differentially phosphorylated in *klkl* and *EFmKL48* mice

**Fig 2:**
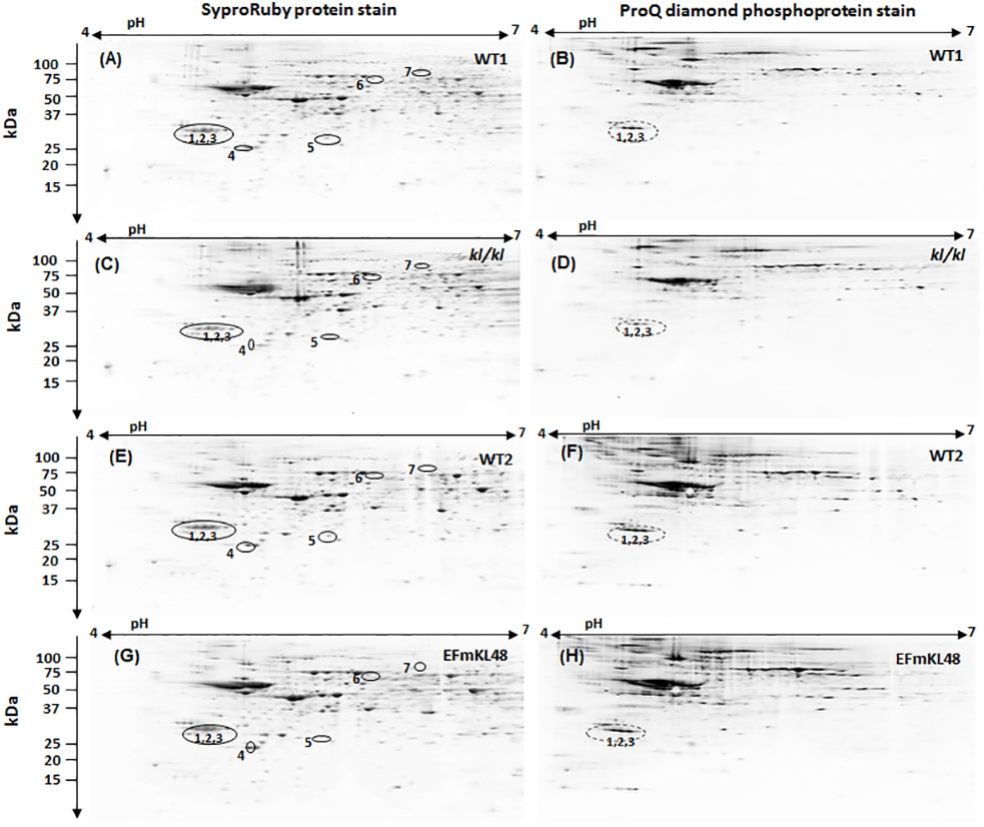
Images of 2D-GE analysis of brain lysates of Klotho KO and Klotho transgenic mice and their WT littermates. (A-H) Representative protein gels stained for phosphorylation (ProQ diamond) and total protein (SyproRuby) for WT (WTI or WT2), Klotho KO (*klkl*), and Klotho transgenic (*EFmKL48*) mice. Circled spots are proteins determined to be either differentially expressed (spots # 4–7) or phosphorylated (spots # 1–3) between WT1 and *klkl* or *EFmKL48* and *klkl*. These are summarized in Table 1.

**Fig 3:**
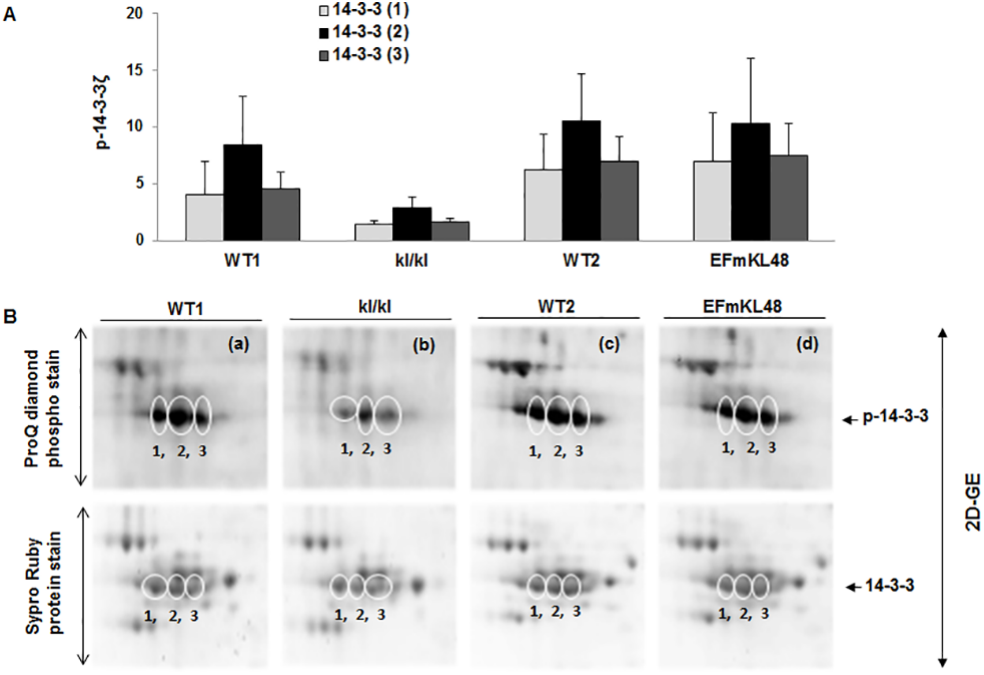
Close-up view of images of 2D-GE extracted from Fig 2. (A) Plots of phosphorylation levels of ProQ diamond stained 14-3-3 proteins. The Pro-Q diamond stained gel images in Fig 3B (a-d) are close-up views of areas of spots in WT1, *kl/kl*, WT2 and EFmKL48 full gels respectively within pI 4.3–4.8. Plots are pooled data from two replicate gels and deviations are shown. Representative ProQ diamond gel images and their corresponding positions in SyproRuby-stained protein gels are shown together to normalize for levels of protein load. Digitized values of stained spots used for the plots are shown in S3 Table.

**Fig 4:**
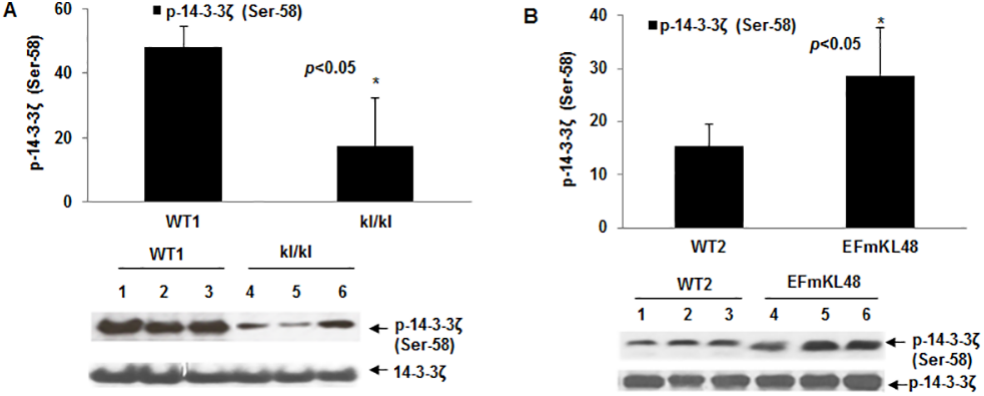
(A) Phospho-14-3-3ζ (Ser-58) levels in WT1 and *kl/kl* mice as determined by Western blot. The averaged phosphorylation level was obtained from three individual mice in each group. Same membrane was stripped after phospho-labeling, and re-probed with total 14-3-3ζ antibody to normalize for protein loading. *p< 0.05 between WT1 and *kl/kl* mice. (B) Western blot profile of Phospho-14-3-3ζ (Ser-58) levels in WT2 and EFmKL48 mice. Details are as described in A above. *p<0.05 between WT2 and EFmKL48. Numerical values from WB used in the plots are provided in S4 Table.

Although our data illustrate that antioxidant and stress proteins, such as peroxidoxin, were differentially expressed between the *klkl* and *EFmKL48* gels using a SyproRuby stain for total protein (Fig 2 and Table 1), a stain of the same gels for phosphoproteins, on the other hand, revealed selective phosphorylation of several proteins that did not match to those spots identified as stress or antioxidant proteins (Fig 2). Particularly interesting were at least three resolved and tandemly positioned protein spots within *p*I range 4.3–4.8 (Fig 2; see close up view in Fig 3B), which were matched to the protein gels and identified by mass spectrometry as 14-3-3 proteins, the specific gene product corresponded to the 14-3-3ζ sub-type. All three phosphoprotein spots were relatively much lower in the *klkl* mice than the levels observed in either the WT or *EFmKL48* mice (Fig 3A). Given that the expression levels of brain 14-3-3ζ remained relatively unchanged as judged by staining for total proteins (Figs 2 and 3B), the relative levels of the endogenous 14-3-3 phosphorylation will reflect the state of cellular activation and seem to correlate with the Klotho expression in each mouse group. The 2D-GE phosphoprotein analysis was further confirmed by Western blotting using as a probe a rabbit polyclonal, anti-phospho-14-3-3ζ (Ser-58) specific antibody (Fig 4). The *klkl* mice had significantly lower basal 14-3-3ζ phosphorylation (Ser-58) (p<0.05) levels as compared to their wild type littermates (Fig 4A). In contrast, when compared to the wild type mice, the *EFmKL48* mice evinced much higher basal phosphorylation levels of 14-3-3ζ (p<0.05) (Fig 4B).

**Table 1:** Summary of seven protein spots of interest identified by mass spectrometry on 2D gels of Klotho mutant and wild type mice.

Spot^*a*^	Protein Information	Accession No.	Peptide count	Protein score	p*I* * (expt/deter)	p*I* * (expt/deter)
1	14-3-3zeta polypeptide	gi4760590	11	402	4.76/4.71	28.8/29.2
2	14-3-3zeta polypeptide	gi4760590	14	578	4.81/4.71	28.8/29.2
3	14-3-3zeta polypeptide	gi4760590	14	634	4.89/4.71	28.8/29.2
4	Peroxiredoxin 2	gi148747558	8	569	5.18/5.2	23.4/21.9
5	Apolipoprotein A-1	gi148693731	13	364	5.73/5.6	26.8/28.9
6	DHLA S-acetyltransferase precursor	gi16580128	11	426	5.82/5.71	67.2/59.3
7	Inner mitochondrial membrane protein	gi70608131	13	187	6.25/6.18	83.8/84.2

^*a*^Spots 1–3 were identified based on their differential phosphorylation [KO mice (klkl) vs. wild type control) Fig 2; spots 4 and 5 were downregulated in klkl vs. wild type; spots 6 and 7 were upregulated in klkl mice.

*expt., experimentally obtained p*I*s and M*r*s are those analyzed from 2D gels; deter., determined pIs and Mrs values are based on primary sequence data and were obtained from literature. DHLA, dihydrolipoamide

### Klotho overexpression or depletion *in vivo* alters the 14-3-3ζ monomer level

**Fig 5:**
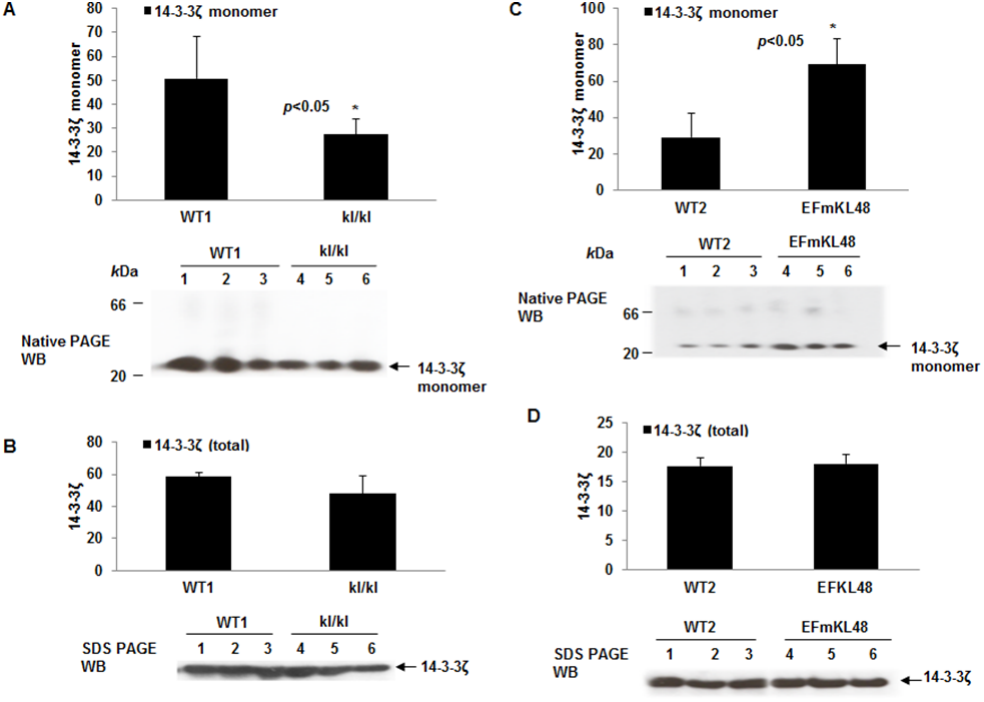
Basal levels of 14-3-3ζ monomer in Klotho KO and Klotho transgenic mice. (A) Plot of native PAGE Western blot of WT1 and *kl/kl* mice probed with 14-3-3ζ antibody. The 14-3-3ζ antibody recognizes a predominant ~30 *k*Da protein band representing the expected size of a monomer. *p< 0.05, between WT1 and *kl/kl*. (B) Replicate samples were separated under SDS-PAGE, electroblotted onto PVDF membrane and probed with same antibody to account for lysate levels of total 14-3-3ζ. (C) Native PAGE Western blot of WT2 verses EFmKL48 performed under identical conditions described for (A). *p< 0.05, between WT2 and EFmKL48. (D) Replicate samples were separated under SDS-PAGE, electroblotted onto PVDF membrane and probed with same antibody to account for lysate levels of total 14-3-3ζ. Digitized values of the WB signals used in the plots are shown in S5 and S6 Tables.

We estimated the relative levels of 14-3-3ζ monomers in the brain lysates derived from *klkl* and *EFmKL48*, and their wild type controls. We found a positive correlation between increased monomer levels and Klotho expression. That is, *EFmKL48* had significantly higher levels of the ~30 kDa 14-3-3ζ monomer as compared to the wild type strains (p<0.05) (Fig 5C), but the *klkl* mice showed decreased brain levels of the monomer relative to its wild type mouse cousins (Fig 5A). For an unbiased estimation of total 14-3-3ζ in the various lysates, we performed Western analysis on replicate samples and observed no significant changes in total 14-3-3ζ protein (Fig 5B & 5D). Since 14-3-3 proteins dimer/monomer changes have been linked to protein-client interactions, it is fair to speculate that increased monomer levels attributed to Klotho overexpression has physiological stabilizing effect on the ASK1 signaling complex. The fact that we can demonstrate a correlation between endogenous 14-3-3ζ phosphorylation and changes in monomer levels implies Ser-58 phosphorylation represents physiologically relevant process for regulating 14-3-3ζ dimerization.

### The brain of Klotho transgenic mouse is protective against MPTP-induced oxidation damage via ASK1 signaling pathway

**Fig 6:**
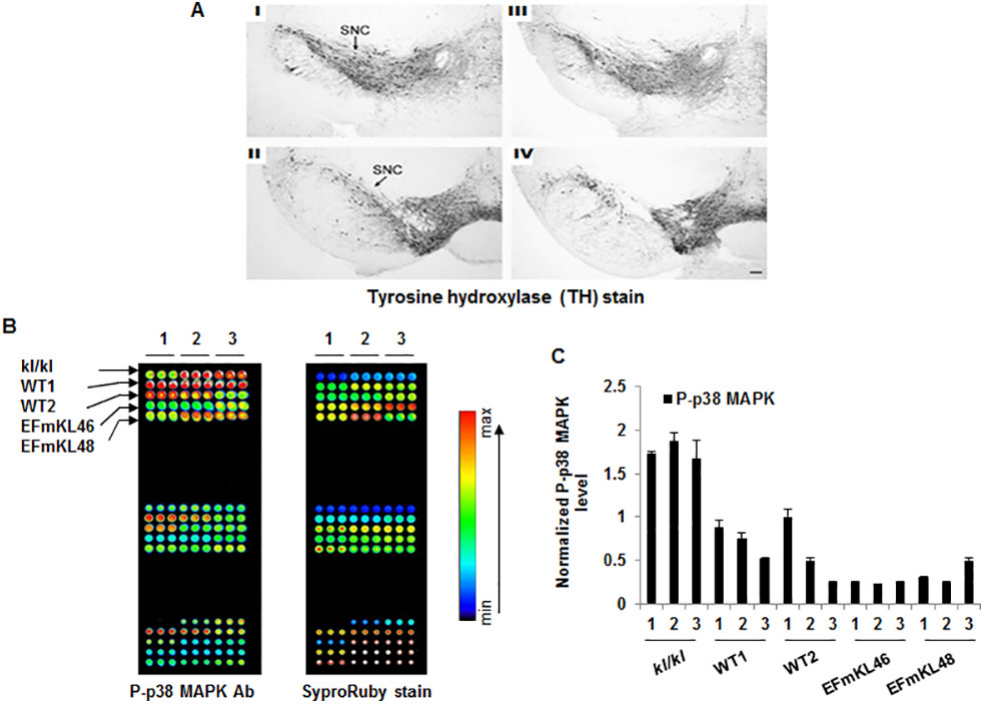
Analysis of dopamine (DA) neurons in substantia nigra (SN) following MPTP treatment of Klotho transgenic (EFmKL48) mice. (A) SN dopamine neurons stained for tyrosine hydroxylase (TH). The SN pars compacta (SNc) neurons which are target for neurodegeneration are shown in MPTP-treated (I and II) and saline control (III and IV) mice. The SNc neurons show no evidence of neurodegeneration following a cumulative dose of 40 mg/kg MPTP. (B-C) Analysis of p38 MAPK activation levels induced by MPTP in the SN. (B) Nitrocellulose backed glass slides arrayed with lysates prepared from SN regions in the brain of various Klotho mouse models and probed with phospho-p38 MAPK antibody and quantum dot (Qdot) secondary antibody detection system (655 nm). Replicate slide was stained for protein with SyproRuby flourescent reagent (Life Technologies). Slides were imaged using the ScanArray^TM^ System (Perkin Elmer). The signal intensity is pseudo-colored, with the lowest intensities in the blue spectrum and the highest intensities in the red-saturated signals appear white. (C) Normalized plots of the phospho-p38 MAPK level. The error bar denotes triplicate array of sample numbered 1–3 and deviations are shown ± SEM. Digitized values of stained spots are shown in S7 Table.

We challenged the potential neuro-protection attributed to Klotho in the transgenic mice strains by administering a specific neurotoxin, MPTP, an oxidant which works through ROS-based mechanisms [16,17]. Since the SN DA neurons, which are vulnerable to degeneration by oxidative stress contain FGFR-1 receptors, a known binding partner for Klotho [18], we hypothesized that Klotho transgenic mice will be less susceptible to MPTP toxicity. We observed a significant loss of striatal dopamine in wild type mice (40%; n = 6-7/group), and a substantial reduction in the number of nigral DA neurons (23%; n = 5/group) relative to the untreated group (Table 2). By contrast, in the *EFmKL48* and *EFmKL46* mouse lines given MPTP, a less significant reduction in striatal DA was observed (26%; n = 5/group and 24%; n = 5/group, respectively for each strain), and virtually negligible loss of nigral DA neurons was observed (6%; n = 5/group and 0%; n = 5/group, respectively) (Table 2). It is important to point out that the reduced sensitivity of the transgenic mice to MPTP toxicity was not due to differences in the metabolism of MPTP. In both wild-type and transgenic mice there were similar levels of MPP+ in the striatum 1.5 hours following an injection of 20 mg/kg MPTP (~30 nmol/g tissue). Furthermore, we analyzed SN DA neuron integrity post-MPTP treatment in the *EFmKL48* mice by staining for tyrosine hydrolase activity relative to saline control mice (Fig 6A). SNc neurons showed no evidence of neurodegeneration following a cumulative dose of 40 mg/kg MPTP. Finally, to determine whether the survival of the DA neurons within the SN of *EFmKL46* or *EFmKL48* mice depended on the ASK1/p38 MAPK signaling pathway, we measured activated p38 MAPK levels in laser microdissected SN regions of the different Klotho mouse lines and their isogenic controls. Using p38 MAPK as a surrogate for the ASK1 signaling activity, we found that the greatest ASK1 activity, as seen by p38 MAPK activation, was within the *klkl* mice and the lowest within the transgenic mouse strains (Fig 6B & 6C). This finding suggests that the DA neurons in the overexpression mice are less vulnerable to ROS-mediated oxidative stress and this protective effect is likely mediated, at least in part, through the stabilization of the ASK1 signaling complex by orchestrated by Klotho signaling.

**Table 2:** Summary of striatal dopamine and substantia nigra (SN) dopaminergic neuron numbers in Klotho overexpressing mice (EFmKL48 or EFmKL46) and their wild type littermates after treatment with MPTP or saline.

^*a*^Mice/Chemical	Dopamine*	%Loss	SN Cell no.*	%Loss	MPP+
WT/Saline	11.92±1.15 (6)	0	4886±240 (6)	0	_
WT/MPTP	7.2±4.7 (7)	40	3779±349 (5)	23	28.5±3.4 (4)
EFmKL46/Saline	9.23±1.53 (4)	0	5078±338 (5)	0	_
EFmKL46/MPTP	6.79±4.25 (5)	26	4758±337 (5)	6	33.2±6.2 (4)
EFmKL48/Saline	12.35±1.51 (3)	0	4649±240 (3)	0	_
EFmKL48/MPTP	9.44±4.3 (5)	24	5080±717 (5)	0	NA

^*a*^Male Klotho overexpressing mice (lines EFmKL48 or EFmKL46) and wild type isogenic mice were administered MPTP at a cumulative dosage of 40 mg/kg. Mice were euthanized 7 days after last injection.

*Student t-test, p<0.05

### Klotho is ubiquitously expressed in various regions of the brain

**Fig 7:**
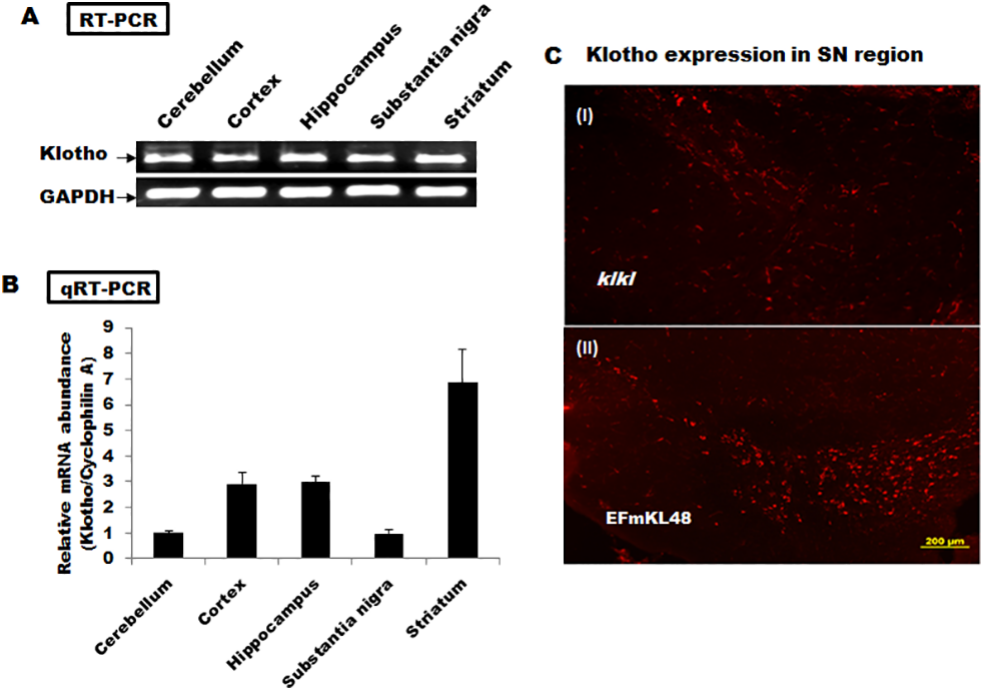
The analyses of Klotho expression from various regions in the brain. (A) RT-PCR profile of Klotho expression relative to GAPDH control. The approximately 630 base pair Klotho product was amplified from normal mouse brain sections using the following specific primers: TGATCAGCGAGCTGGTGCAC (forward), and CCTGTACCTCCAAGTAATCG (reverse). (B) qRT-PCR profile of Klotho expressiondeviations ± SE are shown. (C-D) Immunohistochemical localization of Klotho expression in the SN regions. Details are described in Materials and Methods. Data used for plots in the PCR analyses are shown in S8 Table.

We analyzed the relative levels of Klotho expression in the various regions of the brain using PCR and IHC (Fig 7). Klotho expression was observed in all parts of the brain, with majority concentrated in the striatum based on relative mRNA abundance. This further buttresses the important role for Klotho in cognition function.

## Discussion

Resistance to oxidative stress is identified as an important mechanism for suppressing aging and is evolutionally conserved from worms to mammals [19]. The Klotho protein is an anti-aging hormone described extensively in studies utilizing mouse models that showcase lifespan changes (extended/shortened) depending on gain- or loss-of-function genetic lines; further analysis of these lines demonstrates the role of Klotho in resistance/susceptibility to oxidative stress [3,20,21]. Notwithstanding a few biochemical descriptions that establish the link between oxidative stress status and Klotho’s anti-aging effects, the molecular mechanisms remain largely unknown. Our accumulated data describing the effect of Klotho on ASK1/p38 MAPK stress pathway indicates a complex scheme that depends on posttranslational modifications and multiple protein-protein interactions. Our findings revolve round Trx and 14-3-3ζ interactions with ASK1 and other major signaling proteins composing the ASK1 signaling complex to suppress ASK1 oxidation and dissociation; free ASK1 triggers downstream activation events eventually leading to increased stress levels. Indeed, literature abounds with reports that describe the relationship between endogenous ROS production, ASK1 activation and downstream p38 MAPK activity, and relationship of this signaling to aging and longevity in mouse models [22–24]. But our recent work [6], and the work reported here, all indicate that Klotho has a major effect on these pathways.

From our initial studies of Klotho and oxidative stress, we determined that superoxide dismutase (SOD2) levels were increased via Klotho signaling through Akt activation and FOXO inactivation [3] SOD2 is a well-known anti-oxidant enzyme that converts the oxygen radical to H_2_O_2_; its role in protecting cells, including neurons, against oxidative stress [25,26]. Our earlier profiling of Klotho-stimulated HEK293 cells (unpublished data) revealed several anti-oxidant and stress-related proteins to be upregulated by Klotho; Trx was a robust candidate with an interesting regulatory pattern that impinges upon a well-known stress and apoptotic signaling pathway, the ASK1/p38 MAPK pathway. To determine the involvement of ASK1 signaling complex in regulating stress level in the brain affected by Klotho, firstly, we analyzed clear lysates of brains of mice with differential Klotho expression. Indeed we saw a correlation that mimicked our previous work [6] where a significant increase in the steady-state activation of the pathway was associated with the *kl/kl* mice and the converse result was observed in the overexpression strains. The *kl/kl* mice are models for accelerated aging [2,18] while the transgenic strains exhibit increased lifespan [2,18]. This suggests that in addition to the previously reported effects of Klotho on IGF signaling [27,28], the anti-aging effect of Klotho in the animal models is, in part, mediated through the regulation of anti-oxidant proteins and stress signaling. These data are consistent with our findings of Klotho-dependent SOD2 expression, whereby the activity of the aging suppressor protein may effect a general anti-oxidant and anti-stress response in the brain. This hypothesis therefore complements our previous work describing the effect of Klotho on the liver [6], and extends the relationship between the ASK1 signaling complex and p38 MAPK, and the regulation of this pathway in Klotho responsive models.

The present discovery that Klotho expression in the brain regulates endogenous 14-3-3ζ phosphorylation (Ser-58), followed with increased monomer levels indicates a possible involvement of 14-3-3ζ signaling in pathways activated by Klotho. Although 14-3-3 proteins are known components of the ASK1 signaling complex, their mode of interaction with the complex remains debatable [29–32]. Clearly, our data show positive correlation between Klotho expression and increased 14-3-3ζ phosphorylation (Ser-58) and/or monomerization that warrant further investigation. Nevertheless, the relatively lower basal levels of Ser-58 phosphorylation, and the accompanying low levels of the monomer in the *klkl* animals are consistent with a high basal stress level in a rapidly aging mouse while the levels within the *EFmKL48* strain are consistent with lower oxidative stress in a longer lived and physically robust mouse.

A clear protective effect of Klotho expression in the brain against ROS-mediated damage emerges from our studies on live animals. We tested our hypothesis further using an MPTP-mediated, toxic Parkinsonian mouse model [12,13]. Klotho overexpression was associated with survival of DA neurons in the SN compared to wild type animals. The effects were clear but not dramatic as high MPTP levels or many repeated exposures in wild type animals were lethal; intermediate MPTP concentrations had to be delivered intraperitonially to maximize the comparisons. Laser microdissection and protein microarray analysis of the SN from the different mouse strains demonstrated that the correlations between p38 MAPK phosphorylation and Klotho expression found in the whole brain were maintained focally in the DA neuron-rich nucleus. Local Klotho expression was confirmed for Klotho mRNA by qRT-PCR and immunohistochemical analysis. It is unclear at this time whether the local expression is localized to the DA neurons or the glial cells as we did not assess single cell neuronal mRNA expression due to paucity of material. However, these results do implicate local Klotho signaling in the SN and that the degree of this signaling correlates with local p38 MAPK activation. While we are unable to investigate the ASK1 signaling complex directly in these small SN specimens, if p38 MAPK activation is sufficient as a surrogate, the signaling complex regulation seen in whole brain appears to be operating locally. We believe that this downstream effect of Klotho provides a mechanism for Klotho’s neuroprotective effect as substantiated with a well-known neurotoxic model for Parkinson Disease.

Recently, experiments described Klotho’s involvement in cancer and cardiovascular diseases [33–36]. Considering crucial roles oxidative stress plays in aging and age-associated diseases such as cancer, diabetes and stroke, comprehensive mechanistic understanding of Klotho’s activity in pathways that regulate oxidative stress should be a pressing issue. Indeed, our recent work (unpublished) described a more detailed mechanistic insight into Klotho’s activity within the ASK1/p38 MAPK pathway. Also, it is fair to speculate that Klotho may be involved in other stress associated signal networks such as endoplasmic reticulum stress and GPCR-induced ROS signaling since p38 MAPK activity is regulated in these pathways.

## Supporting Information

S1 Table Digitized values of WB of P-p38 MAPK level in Klotho mice. (XLSX)

S2 Table Digitized values of WB analysis of IP of Trx bound ASK1 in Klotho mice. (XLSX)

S3 Table Digitized values of ProQ diamond vs SyproRuby analysis of 14-3-3 levels in 2D gels. (XLSX)

S4 Table Digitized values of WB analysis of phospho 14-3-3zeta level in brain lysate of Klotho KO and transgenic mice. (XLSX)

S5 Table Digitized values of (i) native PAGE of 14-3-3zeta monomer level and (ii) SDS-PAGE of total 14-3-3zeta in WT vs KO mice. (XLSX)

S6 Table Digitized values of (i) native PAGE of 14-3-3zeta monomer level and (ii) SDS-PAGE of total 14-3-3zeta in WT vs transgenic mice. (XLSX)

S7 Table Digitized values of Qdot analysis of p-38 MAPK phosphorylation in SN of Klotho KO and transgenic (Tg) mice. (XLSX)

S8 Table RT-PCR analysis of Klotho expression in different regions of the brain. (XLSX)
